# Afghan Women’s Use of Violence against Their Children and Associations with IPV, Adverse Childhood Experiences and Poverty: A Cross-Sectional and Structural Equation Modelling Analysis

**DOI:** 10.3390/ijerph18157923

**Published:** 2021-07-27

**Authors:** Jane Ndungu, Rachel Jewkes, Magnolia Ngcobo-Sithole, Esnat Chirwa, Andrew Gibbs

**Affiliations:** 1 Office of Engagement and Transformation, Nelson Mandela University, Port Elizabeth 6001, South Africa; 2 Office of the Executive Scientist, South African Medical Research Council, Pretoria 0001, South Africa; Rachel.Jewkes@mrc.ac.za; 3 Department of Psychology, Nelson Mandela University, Port Elizabeth 6001, South Africa; Magnolia.Ngcobo-Sithole@mandela.ac.za; 4 Gender and Health Research Unit, South African Medical Research Council, Pretoria 0001, South Africa; Esnat.Chirwa@mrc.ac.za (E.C.); Andrew.Gibbs@mrc.ac.za (A.G.)

**Keywords:** Afghan, conflict contexts, corporal punishment, IPV, violence against children

## Abstract

Children who experience violence from a parent are more likely to experience and perpetrate intimate partner violence (IPV) later in life. Drawing on cross-sectional data among married women enrolled in the baseline of a randomized control trial in Afghanistan, we assess risk factors for women’s use of violence against their children, focused on women’s own adverse childhood experiences and experiences of IPV, poverty, poor mental health and gender attitudes. Analysis uses logistic regression and structural equation modelling (SEM). In total 744 married women reported on their use of violence against a child, with 71.8% (*n* = 534) reporting this in the past month. In regression models, their own experiences of witnessing their mother being physically abused, poverty during childhood, current food insecurity, their husband using corporal punishment on their child, current IPV experience, and other violence in the home were all associated with increased likelihood of women reporting corporal punishment. In the SEM, three pathways emerged linking women’s childhood trauma and poverty to use of corporal punishment. One pathway was mediated by poor mental health, a second was mediated by wider use of violence in the home and a third from food insecurity mediated by having more gender inequitable attitudes. Addressing the culture of violence in the home is critical to reducing violence against children, as well as enabling treatment of parental mental health problems and generally addressing gender equity.

## 1. Introduction

Ending violence against children (VAC) is a major health and human rights priority and is part of the Sustainable Development Goals (Target 16.2). Studies have demonstrated that VAC is globally widespread [1,2], yet there remains a limited body of evidence of VAC from humanitarian/conflict settings [3]. In a review of VAC from conflict settings Stark and Landis [3] identified 22 studies, the majority of which were in Africa. They highlighted that there remained very limited research on the drivers of VAC in these contexts, and that the majority of analyses were cross-sectional regression models [3].

Theorisation on the drivers of VAC in conflict and humanitarian settings has hypothesised that VAC may well be higher than in non-conflict settings [4,5]. The elevated rates of VAC may be linked to the introduction of new risk factors, for example exposure to adverse events such as war-related violence, alongside the intensification of existing known risk factors [5,6]. For instance, studies have shown poverty is associated with VAC [7], and in conflict settings livelihoods and survival strategies have often been undermined or destroyed, worsening poverty [5,8]. Parent’s poor mental health is also associated with VAC perpetration [9,10], and mental health may deteriorate further in situations of conflict and displacement, particularly if parents are further exposed to traumatic events, such as conflict, and destruction of homes [11]. Similarly, conflict can lead to more conservative gender norms, and this may impact relationships, including VAC [5,12]. More widely, studies have shown a clustering of inequitable practices in households, whereby violence becomes an acceptable form of social relationship, particularly around men’s attempts to control and discipline women and children [13,14,15]. Despite the theorization about potential pathways between conflict and war trauma and VAC there have been limited studies assessing this.

One form of VAC is corporal punishment, the use of physical violence from parents against their children. Corporal punishment of children has negative physical, psychological and emotional consequences for children [16]. Evidence suggests that children who experience corporal punishment have poorer academic performance, externalizing (e.g., aggression) and internalizing (e.g., depression) behaviours, lower quality social relationships, and poorer mental health throughout the child’s lifespan, and these effects are similar across contexts [17,18,19,20,21].

Afghanistan has experienced over four decades of prolonged conflict, and this has impacted on livelihoods and life trajectories of all Afghans. Previous studies suggest that corporal punishment is common. A trial seeking to reduce violence in schools, found that at baseline 17% of boys and 20% of girls reported experiencing physical punishment at home in the past month [22], while a study by the Central Statistics Organisation and UNICEF [3] reported that three-quarters of children between 2 and 14 years experienced either physical or psychological punishment in the last month at home. Qualitative research from Afghanistan highlighted how the acceptability of violence in the home, potentially driven by stress, as well as a lack of adequate alternative forms of disciplining children, were key drivers of corporal punishment [23].

In this study we examine how among poor women living in Afghanistan the contexts of war and conflict, childhood adverse events, and their own experiences of violence impact on their use of corporal punishment against children. We hypothesise that women who have experienced more violence in their own lives, have been exposed to war traumas, live in households with violence, and have worse mental health are more likely to use corporal punishment.

## 2. Materials and Methods 

### 2.1. Research Design

Data were drawn from the baseline of a randomised controlled trial (RCT) evaluation of the Women for Women International (WfWI) women’s economic and social empowerment intervention [24]. The baseline was conducted between September 2016 and March 2017.

With WfWI we identified five villages located in the provinces of Nangarhar (*n* = 2) and Kabul (*n* = 3), where WfWI were already going to be delivering their intervention during the study period. Together, the population of these two provinces is estimated at 6 million people [25,26]. Villages were selected by WfWI based on geographical, political and social considerations, which were focused on ensuring for safe intervention delivery. A sample of 1461 was recruited.

The eligibility criteria for the participants were twofold (1) the entry criteria for WfWI were that the participant should be poor (earning less than a US$1.25 a day), unemployed, not in school and have not been through the intervention before. WfWI administered their standard processes to identify these women; and (2) for the research, the criteria were that the participant should be able to give informed consent, be willing to participate in the programme and attend all training classes and be between the age of 18 and 45 years. The age group was due to issues of consent for those under 18 years old and reducing the likelihood of mothers-in-law being recruited into the study alongside their daughters-in-law. We also tried to increase the proportion of currently married women in the sample than is usually the case for WfWI programmes, because in Afghanistan it is only possible to ask IPV questions to currently married/previously married women.

All interviews were conducted face-to-face by a trained female interviewer in either Pashto or Dari. Interviewers had prior data collection experience and had additional training for this project. More information on the research design can be found elsewhere [24].

### 2.2. Ethical Considerations

We obtained ethical approval for the study from the Ethics Committee of the Medical Research Council of South Africa (protocol code: EC034-11-2015) and the Institutional Review Board of the Ministry of Public Health of Afghanistan (protocol code: 399302). We also consulted with community elders and men in the villages, prior to commencing the study, to gain their support. Women had very low literacy levels and as such, informed consent was verbally provided with a thumb print on the informed consent form.

### 2.3. Measurement Tools

#### 2.3.1. Corporal Punishment

The main outcome for this analysis is a mother using corporal punishment in the past 4 weeks. For all married women who reported having a child aged 18 or under, we asked: “In the last 4 weeks, how often did you punish your children by giving a slap or beating or otherwise physically punishing them?” Responses were ‘never’, ‘once’, ‘2–3 times’ or ‘4 or more times’. We treated this variable in two ways, in the logistic regression we recoded the responses into no (never) and yes (any positive response), while in the structural equation model we treated it as a discrete variable with 4 levels.

#### 2.3.2. Co-Variates

We assessed a range of socio-demographic characteristics and risk factors for corporal punishment of a child. We asked women their age and recoded it as 18 to 24 and 25 years or older. We also asked participants about their level of education, with responses being ‘none’, ‘madrassa’, ‘primary’ or ‘secondary’ which we coded into formal education (‘primary’ and ‘secondary’) and no formal education (‘none’ and ‘madrassa’).

We assessed poverty through measuring past month household food insecurity using three items from the Household Hunger Scale [27]. An example question was: “In the past 4 weeks, how often was there no food to eat of any kind in your house because of a lack of money?” Responses were ‘never’, ‘rarely’, ‘sometimes’ and ‘often’. We summed the scores and treated the scale as a continuous variable with higher scores indicating more food insecurity (range 3–12, Cronbach α = 0.94).

Gender attitudes were assessed using a 22-item scale developed specifically for the Afghanistan context (Table S1), as existing gender attitude scales did not reflect local gender norms. Questions included “I think the wives in my family should be able to ask a religious scholar about religious issues” and “In this community most people think that girls should go to school” with responses on a 4-point Likert Scale, being, strongly disagree’, ‘disagree’, ‘agree’ and ‘strongly agree’. We summed the scores, with higher scores indicating more gender inequitable attitudes (range 22–88, Cronbach α = 0.87).

Three measures assessed mental health of the sample. Past week post-traumatic stress (PTSD) symptoms were assessed using 16 items from the Harvard Trauma Questionnaire [28]. Sample questions were, “In the past week have you felt as though the event is happening again?”, with not at all, a little, quite a bit and extremely as responses. We summed the scores, with higher scores indicating more PTSD symptoms (range 0–64, Cronbach α = 0.92). Past week depressive symptoms were assessed using the 20-item Center for Epidemiologic Studies Depression Scale (CES-D) [29]. An example question was: “during the past week I felt lonely” and responses being ‘rarely or none of the time’, ‘some of little of the time (1–2 days)’, ‘moderate amount of time (3–4 days)’ and ‘most of all of the time (5–7 days)’. We derived a sum of the scores, with higher scores indicating more past week depressive symptoms (range 0–80, Cronbach α = 0.90). We also measured past four-week suicidal thoughts (ideation), using a single item, with a ‘yes/no’ response option.

Adverse childhood experiences were assessed using 11 items from an adapted version of the childhood trauma questionnaire (Table S1) [30]. Extensive adaptation was required to ensure relevance to Afghan children. This occurred through working with teams in Afghanistan to refine the list of experiences and piloting the questionnaire prior to use. Our working definition of a child was “before marriage” as in Afghanistan that is considered the period of childhood. Three items measured childhood neglect in the context of Afghanistan (e.g., “Before I married I was able to spend time outside the home in fields or in the garden or orchard”), two items childhood hardship (not having enough to eat and having to work to help the family get money), two items on witnessing their mother being physically abused by either her husband, or other family members as a child, two items on childhood emotional abuse, and two items on childhood physical abuse. We could not ask about sexual violence in the study as this was felt by Afghan colleagues to be too sensitive. Responses for each item were, ‘never’, ‘sometimes’, ‘often’ or ‘very often’. Within each form of adverse childhood experience (neglect, hardship, witnessing violence, emotional abuse, physical abuse) we coded women reporting any positive response to having experienced that form of childhood adversity (Range 12–36, Cronbach α = 0.65). In the structural equation model we treated this as a continuous variable where we summed all items.

We assessed two forms of violence women may have experienced. Among women we assessed past year physical intimate partner violence (IPV) experience using five items from the World Health Organisation’s instrument [31]. Questions asked included, “In the past 12 months how many times has your husband slapped you or thrown something at you which could hurt you?” With responses being ‘never’, ‘once’, ‘few times’ or ‘many times’. We coded these into yes and no, with a positive response to any item being coded into having experienced physical IPV. We also asked about violence from their mother-in-law in the past 12 months (yes/no) and coded any positive response to yes.

Because of the strong overlap between the different forms of familial violence (physical IPV and family violence) we recoded these into a four-level variable: experiencing none; experiencing violence from a mother-in-law, but not others; experiencing physical IPV but not violence from a mother-in-law; and experiencing both physical IPV and violence from a mother-in-law.

Exposure to war and traumatic events were measured using five questions asking whether the woman had in her lifetime ever witnessed the killing of a family member or friend, the death of a stranger or someone she knew, or a rocket attack or bomb falling or an armed attack on someone; and whether she had ever been or felt she was close to death, or in a disaster like a fire, flash flood, or earthquake. Responses were dichotomous (yes/no). In the logistic regression we recoded responses into: 0 events, 1 or 2 events, and 3 or more events, and in the structural equation modelling (SEM) we used it as a score.

We also asked whether their husband had used corporal punishment on the child, using one item, “in the last 4 weeks, how often did your partner or one of his relatives punish your children by smacking or beating them?” Responses were ‘never’, ‘once’, ‘2–3 times’ and ‘4 or more times’. We coded these into ever and never.

### 2.4. Statistical Analysis

To begin with, we computed descriptive statistics and present percentages (%) and sample size (n’s) or means (M) and 95% confidence intervals (95% CIs), and standard deviations (SDs). We then conducted bivariate analysis, comparing those who used corporal punishment on their children to those who did not, on all risk factors. For categorical variables, we assessed associations using Pearson’s Chi-Squared tests, and for continuous variables we used *t*-tests. We present percentages/means, 95% confidence intervals (95% CI) and *p*-values as appropriate.

We then computed multivariable regression analysis to assess associations between use of corporal punishment and risk factors. In the regression model, we included all candidate variables from the descriptive analysis. We present adjusted odds ratios (adj. OR), 95% confidence intervals and *p*-values. These analyses were conducted in STATA/IC13.0 and accounted for the clustered nature of the sample.

We also conducted SEM in MPlus to assess pathways to corporal punishment of children. To build the SEM we first developed a theoretical model of the pathways based on prior research and the multivariable logistic regression. We hypothesised that poverty and traumatic exposures were directly associated with corporal punishment, and that there were mediated pathways via poor mental health, gender inequitable attitudes and family violence. We created one latent variable, mental health, which we included in the variables for depression, PTSD and suicidal ideation, and fitted it assessing goodness of fit. Our theoretical model was based on the logistic regression, previous research and our hypotheses. Based on our theoretical model, we first regressed each variable to one another on the pathways (for example poverty to depression) retaining only those which were significant linear or logistic regression as appropriate and retained pathways where *p* < 0.05. We then fitted the structural model and used backwards elimination to remove non-significant (*p* > 0.05) pathways. We assessed goodness of fit, and then varied error terms were indicated and theoretically plausible. We understood a good fit as CFI > 0.95, TLI > 0.95 and RMSEA < 0.05.

Due to having a combination of categorical and discrete measures in the models as well as the presence of missing data in some of the measures, we used weighted least squares mean and variance estimators (WLSMV) with theta parametisation, to estimate the simultaneous equations [32]. We used the Chi-square difference testing to test the significance of removing variables in the model.

We report unstandardised and standardised scores for the model coefficients with their 95% confidence limits. Analyses were carried out in Mplus 8.6 software package (Muthen and Muthen, Los Angeles, CA 90066, USA).

## 3. Results

### 3.1. Demographic Description of the Sample

Table 1 presents the descriptive statistics. The past month prevalence of corporal punishment was 53.9%. More than four fifths of the women (88.7%) were over 25 years of age. The vast majority of women lacked formal education (86.1%). Experiences of adverse childhood experiences were common. Specifically, over half of the women (69.8%) had experienced neglect as children, just over a third (39.9%) had experienced hardship as a child, over a fifth (22.7%) had, as a child, witnessed their mother being physically abused, almost a fifth (17.3%) had experienced childhood emotional violence and 6.8% reported having experienced childhood physical violence (Table 1).

Overall, 28.5% reported experiencing violence in the household. Just over one in 20 (5.2%) reported having experienced IPV but not family abuse in the past year, 19.0% had experienced past year IPV but not family abuse and just under one in 20 (4.3%) had experienced both IPV and family abuse in the past year. Almost a tenth (6.9%) had experienced suicidal ideation. Two thirds (66.1%) of the women had been exposed to specific war related events. Just over a half (56.1%) had been exposed to one or two war events, and a tenth (9.7%) had been exposed to three or more war events.

Over half (53.9%) of the women had partners who also used corporal punishment on their children (Table 1).

### 3.2. Bivariate Analysis

We present bivariate descriptive analysis assessing risk factors for corporal punishment in Table 1. Compared to women who did not use corporal punishment on their children, a greater proportion of those who reported using corporal punishment on their children were older (*p* = 0.015); and had higher mean scores for food insecurity (*p* < 0.001), gender inequitable attitudes (*p* < 0.001), PTSD symptoms (*p* < 0.001) and depressive symptoms (*p* < 0.001), and had suicidal ideation (*p* < 0.001).

Experiences of adverse events as children were associated with corporal punishment of children (Table 1), with a greater proportion of those who reported using corporal punishment on their children reporting experiencing childhood physical violence (*p* < 0.001), had, as a child, witnessed their mother being physically abused (*p* < 0.001) and childhood emotional violence (*p* < 0.001). A smaller proportion of those who reported using corporal punishment on their children reported childhood neglect (*p* < 0.001).

Descriptively, the proportion of women using corporal punishment on their children was greater among those reporting both IPV and experience of violence from mother-in-law (*p* < 0.001) and a higher proportion reported their partner also used corporal punishment on their child (*p* < 0.001).

### 3.3. Multivariable Regression Analysis

Multivariable regression assessing risk factors for corporal punishment of a child is presented in Table 2. In the multivariable regression, corporal punishment of a child was associated with worse food insecurity (adj. OR 1.10, *p* = 0.017), greater gender inequitable attitudes (adj. OR 1.14, *p* < 0.001), witnessing the mother being physically abused as a child (adj. OR 2.50, *p = 0*.001), experiencing hardship as a child (adj. OR 1.81, *p* = 0.003), experiencing IPV only but no violence from mother-in-law (adj. OR 3.55, *p* < 0.001), and having a husband who also uses corporal punishment on their children (adj. OR 3.26, *p* < 0.001). Experiencing neglect as a child was associated with a reduction in the likelihood of corporal punishment of their own children (adj. OR 3.09, *p* = 0.001).

### 3.4. Structural Equation Model

**Figure 1:**
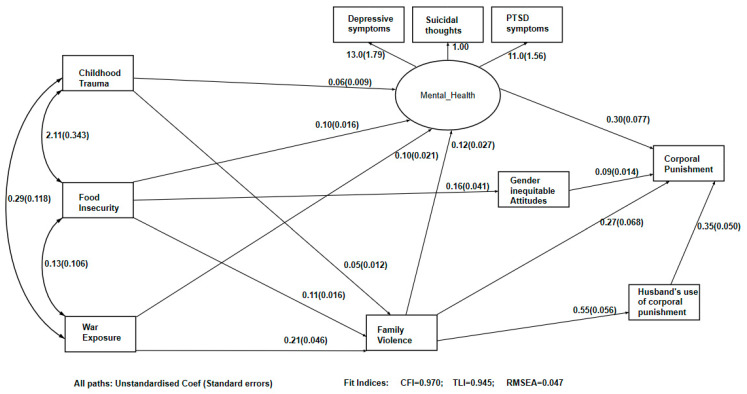
Structural equation model showing pathways from adverse childhood experiences, food insecurity and war exposure to mother using corporal punishment on the child.

Table 3 and Figure 1 show the SEM of pathways to corporal punishment of a child. The SEM had good goodness of fit statistics (Figure 1). Adverse childhood experiences, exposure to war impacts and food-insecurity were all exogenous variables, and there were three sets of mediated pathways from these to corporal punishment: one via poor mental health, a second via gender inequitable attitudes, and a third via other violence in the household.

All three exogenous variables were associated with an increase in poor mental health, and women’s increased poor mental health was associated with an increased use of corporal punishment. A second pathway mediated the relationship from food insecurity to corporal punishment via gender inequitable attitudes, where higher levels of food insecurity increased gender inequitable attitudes and they in turn increased corporal punishment. The final mediated pathway was family violence, whereby food insecurity, childhood adverse events and war exposure all increased the likelihood of more severe forms of violence the woman experienced. This was directly associated with increased corporal punishment, and indirectly through the woman’s husband also using corporal punishment on the child.

## 4. Discussion

Among poor married women in Afghanistan experiencing multiple forms of challenges including poverty, and prior histories of adverse events during childhood and linked to war, as well as their own experiences of violence and poor mental health, corporal punishment of children was common, with just over half reporting using this in the past month. This analysis highlights how women’s use of corporal punishment against their children is located in contexts of adversity and challenge. Importantly our work, while reflecting prior work on drivers of parental corporal punishment against children [14], extends this to conflict settings, and how exposure to conflict-related adverse events further exacerbates known risk factors for corporal punishment.

As we hypothesized, we found women who had experienced violence from multiple settings, including their husband and family, in childhood were more likely to use corporal punishment against their own children. Prior studies have clearly described the clustering and overlap between different forms of household violence, including intimate partner violence, mother-in-law violence, and sibling violence [14,33,34]. Qualitative research has suggested that this is associated both with the acceptability of violence in household units, but also how gender inequalities play out in the household, with men using violence as a way to discipline women and children [13]. Addressing violence at the household level may be an important strategy for addressing corporal punishment.

The analysis also demonstrated how poverty was associated with the use of violence by women against their children. There was no direct pathway between poverty and corporal punishment, rather poverty exacerbated a range of factors, including poor mental health, gender inequitable attitudes and increased the likelihood of experience of other forms of violence in the household. Studies have previously described poverty as a risk factor for corporal punishment of children [7,14,35], and some have suggested that in contexts of poverty the use of corporal punishment is a way for parents to try and maintain respectability, where other sources of respectability are not achievable [35]. Other studies have found that poverty increases parental stress, which negatively impacts on parental emotional regulation, creating risk for corporal punishment [36,37].

In the SEM and logistic regression, gender inequitable attitudes were associated with corporal punishment, and in the SEM poverty was associated with increased gender inequitable attitudes. The pathway from poverty to gender inequitable attitudes has been shown in other studies [14,38]. Gender inequitable attitudes, particularly the acceptability of violence in everyday social relationships, including against children, is an important driver of corporal punishment [22], and important to target in any interventions to reduce corporal punishment. In the SEM, gender inequitable attitudes were not associated with other forms of violence in the home, although this may be because we assessed women’s individual attitudes, while we assessed other family members use of violence against women, and these two may not necessarily be linked.

Our analysis showed no direct pathway between childhood adversity and corporal punishment of children, rather all pathways were mediated by poor mental health and other forms of violence in the household. Previous research has also demonstrated a relationship between experience of childhood adversity and subsequent corporal punishment and suggested that this may be linked to learnt behaviour [7]. While this may certainly be one aspect, it is likely that there is also an aspect of learning how violence can achieve certain aims including ensuring your desires are met [39]. Adverse childhood experiences were also associated with poorer mental health, and increased levels of violence against the woman from other household members. This once again reinforces the clustering of violence in households as an important issue to address.

In contrast to previous research [14,40], in our analysis our measure of emotional neglect in childhood was associated with a reduced likelihood of using corporal punishment. Our measure of neglect included items such as being able to spend time outside the home in fields or gardens, and this may be indicative of a more liberal upbringing in this context, rather than neglect. In addition, being outside of the home, may have reduced the amount of disciplining that children experienced from adults. Further research is needed to understand how to conceptualise neglect in settings such as Afghanistan, where raising children in contexts of ongoing war may reshape what neglect looks like.

Prior research has highlighted the importance of women’s poor mental health in their use of violence against children, and this was seen in our analysis too. Importantly, poor mental health was shaped by women’s own experiences of poverty, and war exposure and childhood adverse experiences. Prior studies have often focused on depressive symptoms as the causal risk factor [10,14], with fewer focusing on PTSD [9]. Studies on depressive symptoms as a risk factor for corporal punishment have found inconsistent results [10,14]. There are a range of potential pathways through which maternal PTSD may be associated with greater likelihood of corporal punishment. These include greater impulsivity, which has been associated with the perpetration of violence in multiple studies [9]. Other studies have highlighted how parents with PTSD may be less emotionally available, and may perceive children’s behaviour more negatively [41]. In addition, children who are raised by parents with PTSD may have more behavioural problems, which where the use of corporal punishment is accepted, may lead to them experiencing corporal punishment more often [42].

### Limitations

This study has a number of limitations. Due to the context, and upon advice from the Afghan study staff, we were unable to ask about sexual violence in adulthood or in childhood, which may be associated with corporal punishment of children. We did not ask about children’s ages and given that corporal punishment is more commonly experienced by younger children, we cannot be sure how this would have changed our findings. We used a significantly modified childhood trauma questionnaire, which was needed because of the context in which Afghan women grew up, however we did not do a formal test of reliability and validity. Our sample was relatively homogenous in terms of age of mothers and educational levels of mothers, and this further limits our understanding of how these may impact on corporal punishment. Our data were obtained from a non-random sample; thus, we cannot generalise the findings, although it is unclear how this would change our analysis. The data are cross-sectional, and thus a number of drivers may be bi-directional in the analysis. Interviews were conducted by a team of trained field-staff in spaces where audio-privacy could be assured, but there is often a reluctance to speak about ‘issues in the home’ to researchers.

## 5. Conclusions

Our analysis showed women’s use of corporal punishment against their children was common among poor women in Afghanistan and was associated with women’s own experiences of adverse experiences as children, and in later life, and particularly violence directed to them in their household, poor mental health, as well as the high levels of poverty women experienced. Women’s experiences of war trauma were central in exacerbating these risk factors. Strategies to reduce corporal punishment have typically focused on ‘teaching’ parents different styles of disciplining children [43,44], yet our analysis suggests that for very marginalized women, experiencing multiple challenges, effective strategies to prevent corporal punishment need to go beyond this, to address the underlying causes of the use of violence. First, interventions need to be embedded within wider poverty reduction initiatives. Second, given the importance of women’s poor mental health as a mediating factor, addressing women’s poor mental health could be an important strategy. Third, our analysis clearly showed how women’s use of corporal punishment against their children was linked to a clustering of the use of violence at the household level and addressing corporal punishment needs to be undertaken within a holistic programme which addresses all forms of violence in the household. A recent intervention in Tajikistan focusing on reducing IPV through a family intervention, also found a reduction in corporal punishment of children [45], highlighting how all forms of violence are modifiable. Finally, interventions to reduce corporal punishment need to be firmly located within a framework of addressing gender inequalities. Gender inequalities are critical for the maintenance of attitudes supportive of the use of violence. Addressing corporal punishment is critical for ensuring we achieve the Sustainable Development Goals and for creating more peaceful societies.

## Figures and Tables

**Table 1:** Socio-demographic descriptive and bivariate descriptive statistics for using corporal punishment on the child.

Variable	*n*	% (*n*)/Mean, SD (95% CI)	Mother Using Corporal Punishment on Their Child		
Socioeconomic Status			No% (*n*)/Mean	Yes% (*n*)/Mean	*p*-Value
Age	855				
18 to 24		11.4 (97)	14.2 (56)	8.9 (41)	0.015
25+		88.7 (758)	85.8 (338)	91.1 (420)	
Level of education					
No formal education	853	86.1 (734)	84.7 (333)	87.2 (401)	0.303
Any formal education		14.0 (119)	15.3 (60)	12.8 (59)	
**Food insecurity > more**	854	5.5, 2.85 (5.27, 5.66)	4.8	6.1	<0.001
**Gender attitudes > more gender inequitable attitudes**	854	21.8, 3.15 (21.67, 22.10)	19.1	20.4	<0.001
**Mental health**					
Posttraumatic Stress Disorder (PTSD) > more	857	25.7, 8.31 (25.11, 26.23)	7.73	11.4	<0.001
Depression > more	854	15.7, 10.07 (14.98, 16.33)	13.7	17.4	<0.001
Suicidal ideation (past 4 weeks)	851				
No		93.1 (792)	97.5 (385)	89.3 (407)	<0.001
Yes		6.9 (59)	2.5 (10)	10.8 (49)	
**Childhood trauma**					
Childhood physical violence	854				
No		93.1 (796)	97.2 (383)	89.8 (413)	<0.001
Yes		6.8 (58)	2.8 (11)	10.2 (47)	
As a child, witnessing mother being physically abused	855				
No		77.3 (661)	87.1 (343)	69.0 (318)	<0.001
Yes		22.7 (194)	12.9 (51)	31.0 (143)	
Experiencing childhood emotional violence	855				
No		82.7 (707)	89.6 (353)	76.8 (354)	<0.001
Yes		17.3 (148)	10.4 (41)	23.2 (107)	
Experiencing hardship as a child	855				
No		60.1 (514)	71.3 (281)	50.5 (233)	<0.001
Yes		39.9 (341)	28.7 (113)	49.5 (228)	
Experiencing neglect as a child	854				
No		30.2 (258)	21.9 (86)	37.3 (172)	<0.001
Yes		69.8 (596)	78.1 (307)	62.7 (289)	
**Experience of trauma in adulthood**					
Family violence	793				
None		71.5 (567)	79.0 (567)	0	<0.001
Violence from mother-in-law		5.2 (41)	0	54.7 (41)	
Intimate partner violence (IPV)		19.0 (151)	21.0 (151)	0	
Both IPV and violence from mother-in-law		4.3 (34)	0	45.3 (34)	
War Exposure	886				
None		34.2 (268)	35.3 (251)	23.3 (17)	0.09
Exposure to one or two		56.1 (440)	55.4 (394)	63.0 (46)	
Exposure to three or more events		9.7 (76)	9.3 (66)	13.7 (10)	
**Corporal punishment**					
Partner uses corporal punishment on the child	854				
No		56.3 (481)	71.8 (282)	43.2 (199)	<0.001
Yes		43.7 (373)	28.2 (111)	56.8 (262)	
Mother uses corporal punishment on the child (outcome variable)	855				
No		45.1 (394)			
Yes		53.9 (461)			

**Table 2:** Multivariable regression model for risk factors for mother using corporal punishment on the child.

Mother Uses Corporal Punishment on the Child (*n* = 711)	Adj. OR	*p* > |t|	[95% Conf. Interval]	
			Low	High
**Socioeconomic status**				
Age				
18–24	ref	ref	ref	ref
25+	0.98	0.86	0.76	1.26
Level of education				
No formal education	ref	ref	ref	ref
Any formal education	0.97	0.812	0.76	1.24
**Food insecurity**				
>more	**1.1**	**0.017**	1.02	1.19
**Gender attitudes**				
>more gender inequitable attitudes	**1.14**	**<0.001**	1.07	1.21
**Mental health**				
Posttraumatic Stress Disorder (PTSD)				
>more	1.04	0.089	0.10	1.08
Depression				
>more	0.99	0.661	0.97	1.02
Suicidal ideation (past 4 weeks)				
No	ref	ref	ref	ref
Yes	1.63	0.327	0.62	4.30
**Childhood trauma**				
Childhood physical violence				
No	ref	ref	ref	ref
Yes	1.34	0.594	0.46	3.91
As a child, witnessing mother being physically abused				
No	ref	ref	ref	ref
Yes	**2.50**	**0.001**	1.55	4.04
Experiencing childhood emotional violence				
No	ref	ref	ref	ref
Yes	1.076	0.807	0.60	1.95
Experiencing hardship as a child				
No	ref	ref	ref	ref
Yes	**1.81**	**0.003**	1.23	2.67
Experiencing neglect as a child				
No	ref	ref	ref	ref
Yes	**0.49**	**0.001**	0.33	0.73
**Experience of trauma in adulthood**				
Family violence				
None	ref	ref	ref	ref
Violence from mother-in-law	1.78	0.158	0.80	3.95
Intimate partner violence (IPV)	**3.30**	**<0.001**	1.90	5.73
Both IPV and violence from mother-in-law	1.66	0.368	0.55	5.05
War exposure				
None	Ref	Ref	Ref	ref
Exposure to one or two event(s)	1.24	0.288	0.84	1.83
Exposure to three or more events	1.02	0.951	0.47	2.23
**Corporal punishment**				
Partner uses corporal punishment on the child				
No	ref	ref	ref	ref
Yes	**3.09**	**<0.001**	2.15	4.45

**Table 3:** Structural equation model showing pathways from childhood adverse events, poverty and war exposure to corporal punishment.

Pathway	Unstandardised Coef. (95% CI)	Standardised Coef. (95%CI)	*p*-Value
Mental Health -> Corporal Punishment	0.30 (0.15–0.45)	0.16 (0.08–0.23)	<0.001
Family Violence -> Corporal Punishment	0.27 (0.14–0.40)	0.23 (0.12–0.35)	<0.001
Gender inequitable attitudes -> Corporal Punishment	0.09 (0.06–0.12)	0.22 (0.15–0.29)	<0.001
Partner using Corporal Punishment on the child -> Corporal Punishment	0.35 (0.25–0.45)	0.32 (0.24–0.41)	<0.001
Family Violence -> Partner using Corporal Punishment on the child	0.55 (0.43–0.66)	0.51 (0.44–0.59)	<0.001
Childhood Trauma -> Mental Health	0.06 (0.04–0.07)	0.28 (0.24–0.33)	<0.001
War Exposure -> Mental Health	0.10 (0.05–0.14)	0.15 (0.10–0.20)	<0.001
Food Insecurity -> Mental Health	0.10 (0.07–0.13)	0.42 (0.36–0.49)	<0.001
Family Violence -> Mental Health	0.12 (0.07–0.18)	0.20 (0.13–0.27)	<0.001
Childhood Trauma -> Family Violence	0.05 (0.03–0.07)	0.16 (0.08–0.23)	<0.001
Food Insecurity -> Family Violence	0.11 (0.08–0.14)	0.28 (0.20–0.36)	<0.001
War Exposure -> Family Violence	0.21 (0.12–0.30)	0.21 (0.12–0.29)	<0.001
Food Insecurity -> Gender inequitable attitudes	0.16 (0.08–0.24)	0.15 (0.08–0.22)	<0.001
**Covariances**			
Family violence with suicidal thoughts	0.62 (0.46–0.78)	0.62 (0.46–0.78)	<0.001
Childhood trauma with Gender inequitable attitudes	−1.36 (−2.04–−0.68)	−0.13 (−0.19–−0.06)	<0.001
Childhood trauma with Food insecurity	2.11 (1.44–2.78)	0.21 (0.15–0.27)	<0.001
Childhood trauma with war exposure	0.29 (0.06–0.52)	0.08 (0.02–0.14)	0.015
War exposure with Food insecurity	0.13 (−0.08–0.34)	0.04 (−0.03–0.11)	0.233
**RMSEA = 0.047, CFI = 0.97, TLI = 0.95**			
**Estimator: Weighted Least Squares Mean and Variance Estimators (WLSMV) with theta Parameterization**			
**Link function: Probit**			

## Data Availability

Data are available in a public, open access repository. De-identified data sets for the project are available from http://medat.samrc.ac.za/index.php/catalog/WW (accessed on 1 July 2021) managed by the South African Medical Research Council.

## Supplementary Materials

The following tables are available online at https://www.mdpi.com/article/10.3390/ijerph18157923/s1. Table S1. Scales used in the WfWI trial in Afghanistan.
