# Supplementary material for: Afghan Women’s Use of Violence against Their Children and Associations with IPV, Adverse Childhood Experiences and Poverty: A Cross-Sectional and Structural Equation Modelling Analysis
Source: Int J Environ Res Public Health. 2021 Jul 27;18(15):7923. doi: 10.3390/ijerph18157923 (PMC8345444; doi:10.3390/ijerph18157923)
Supplement: Supplementary file 1 [file ijerph-18-07923-s001.zip › ijerph-1241379-supplementary.pdf]

Scales used in the WfWI trial in Afghanistan

SECTION 2 ATTITUDES ABOUT RELATIONS BETWEEN MEN AND WOMEN					
The next set of questions are about your views on life and particularly on relations between men and women in society. There are no right or wrong answers – we are just interested in what you think.<br><br>For each of the following statements please say answer whether you strongly agree, agree, disagree or strongly disagree with the following statements:					
201		STRONGLY DISAGREE	DISAGREE	AGREE	STRONGLY AGREE
A	In this community many people think that girls should go to school	1	2	3	4
B	I think girls in my family should go to school	1	2	3	4
C	In this community many people think that husbands should give permission to the wives to go to the clinic	1	2	3	4
D	I think the husbands in my family should give permission to give their wives to go to the clinic	1	2	3	4
E	In this community many people think that husbands should listen to their wives’ opinion on schooling	1	2	3	4
F	I think the husbands in my family should listen to their wives’ opinion on schooling	1	2	3	4
G	In this community many people think that wives should have a say in how money is spent	1	2	3	4
H	I think the wives in my family should have a say in how money is spent	1	2	3	4
I	In this community many people think that wives should be able to ask a religious scholar about religious issues	1	2	3	4
J	I think the wives in my family should be able to ask a religious scholar about religious issues	1	2	3	4
K	In this community many people think that husbands should respect the opinion of their wives on matters related to income generating work	1	2	3	4
L	I think the husbands in my family should respect the opinion of their wives on matters related to income generating work	1	2	3	4
M	In this community many people think that a husband should be kind and care about the happiness of women in his family	1	2	3	4
N	I think husbands in my family should be kind and care about the happiness of women in the family	1	2	3	4

O	In this community many people think that wives should always obey their husband	1	2	3	4
P	I think that the wives in my family should always obey their husbands	1	2	3	4
Q	In this community many people think that if a wife in our family does something wrong her husband has the right to punish her	1	2	3	4
R	I think that if a wife in my family does something wrong her husband has the right to punish her	1	2	3	4
S	In this community many people think that a wife who does things that are wrong should be beaten to correct her behaviour	1	2	3	4
T	I think that a wife in my family who does things that are wrong should be beaten to correct her behaviour	1	2	3	4
U	In this community many people think that it is good to beat a young wife to teach her how to behave properly	1	2	3	4
V	I think it is a good thing for a young wife in my family to be beaten to teach her how to behave properly	1	2	3	4

	**SECTION 6: CHILDHOOD AND OTHER LIFE EXPERIENCES**				
	**THESE QUESTIONS ARE PHRASED AS "BEFORE YOU WERE MARRIED" BUT IF THE WOMAN HAS NOT BEEN MARRIED, PLEASE REPHRASE EACH AS "BEFORE YOU WERE 18…"**				
	Thank you for answering these questions. The questionnaire will be finished soon. We would just like to ask you some questions about **your childhood before you married** and other experiences you have had in your life. First we have a series of statements about your childhood. For each we would like to know if they never, sometimes, often or very often happened				
601		NEVER	SOMETIMES	OFTEN	VERY OFTEN
A	Before I married I did not have enough to eat	1	2	3	4
B	Before I married I lived in different households at different times	1	2	3	4
C	Before I married I saw or heard my mother being beaten by her husband	1	2	3	4
D	Before I married I was told I was lazy or stupid or weak by someone in my family	1	2	3	4
E	Before I married I saw or heard my mother being beaten by my mother-in-law or another person in the family	1	2	3	4
F	Before I married I was insulted or humiliated by someone in my family in front of other people	1	2	3	4

G	Before I married I was beaten at home with a belt or stick or whip or something else which was hard	1	2	3	4
H	Before I married I had to work at home to help the family get money	1	2	3	4
I	Before I married one or both of my parents was not able to take care of me	1	2	3	4
J	Before I married I was beaten so hard at home that it left a mark or injured me	1	2	3	4
K	Before I married I was able to spend time outside the home in fields or in the garden or orchard	1	2	3	4
L	Before I married I was often afraid we would be killed or injured in the conflict or war	1	2	3	4
